# Supplementary material for: RppH can faithfully replace TAP to allow cloning of 5′-triphosphate carrying small RNAs
Source: MethodsX. 2019 Jan 29;6:265–72. doi: 10.1016/j.mex.2019.01.009 (PMC6369235; doi:10.1016/j.mex.2019.01.009)

Supplementary figure 1

Rpph-Replicate1

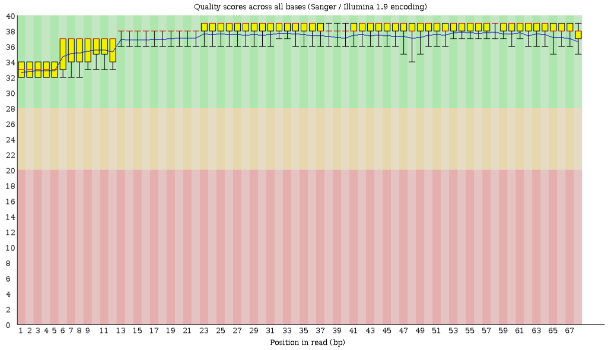

TAP-Replicate1

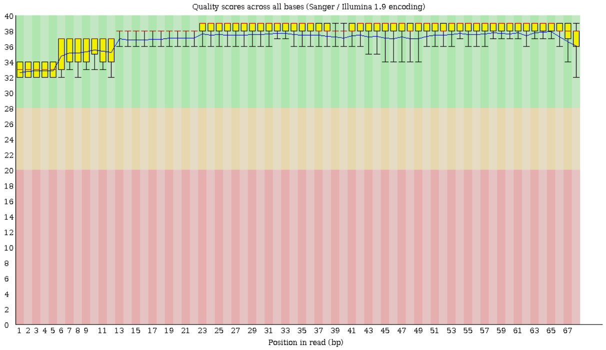

Rpph-Replicate2

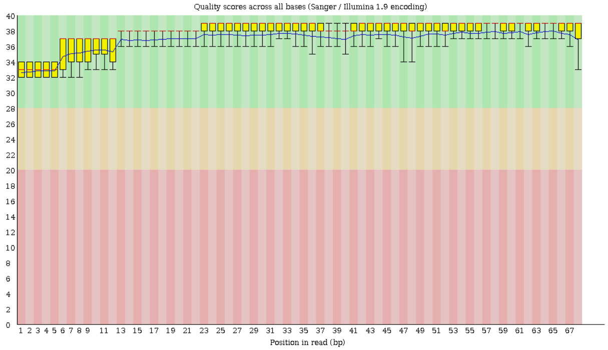

TAP-Replicate2

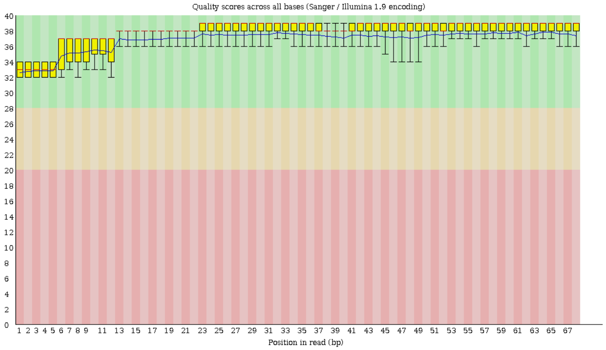

Rpph-Replicate3

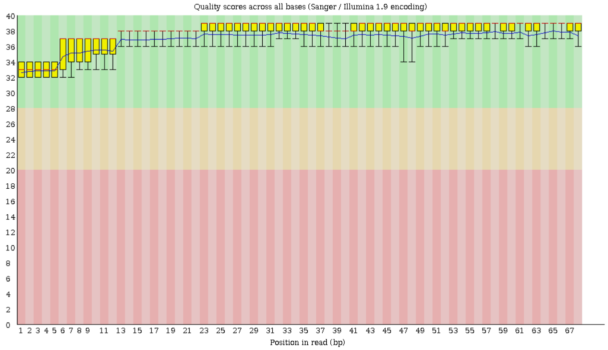

TAP-Replicate3

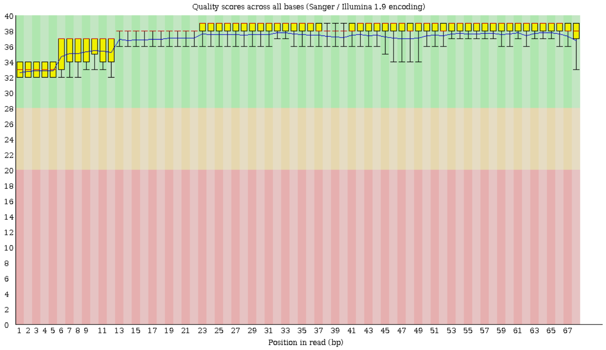

Supplementary figure 2

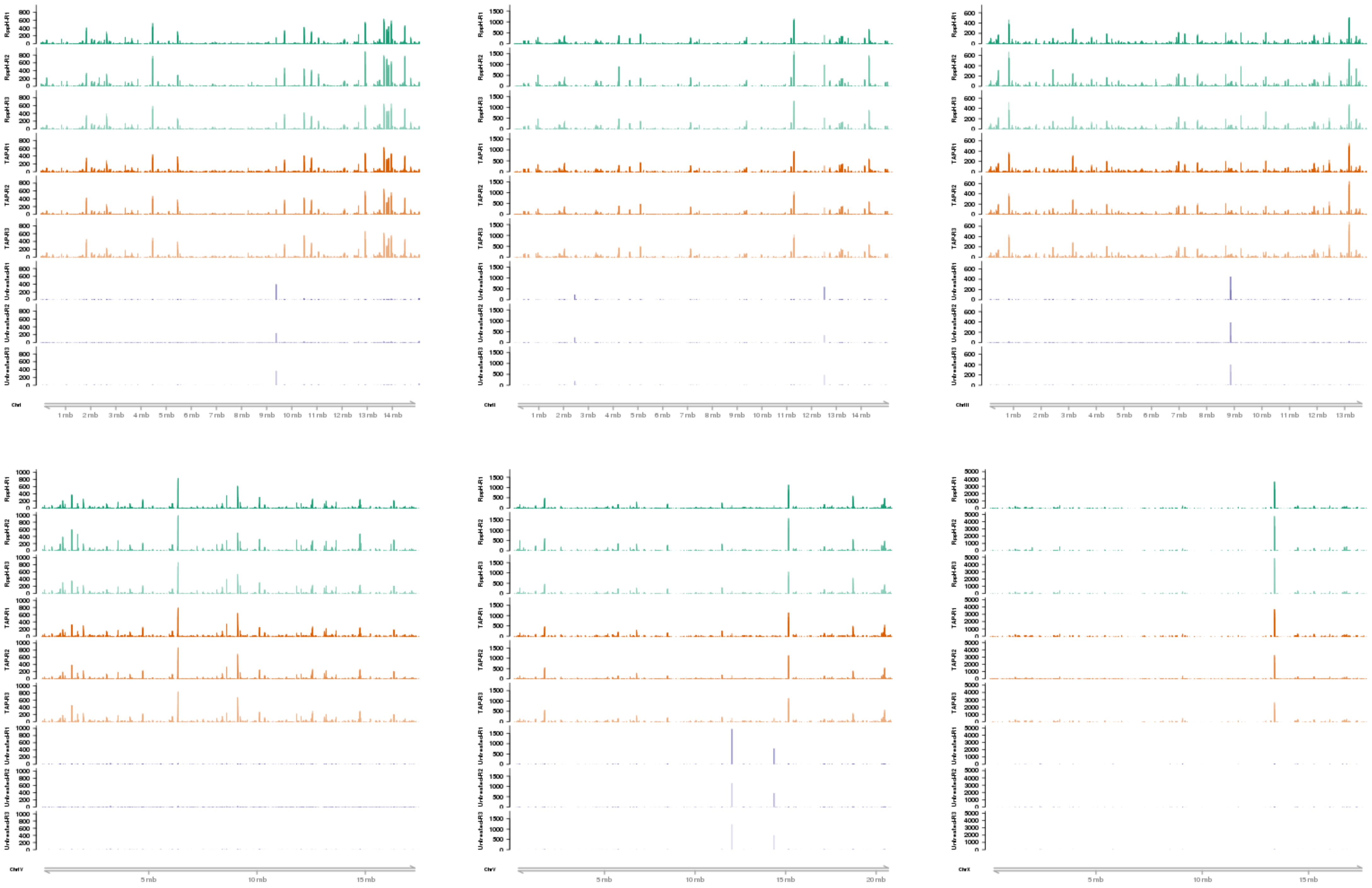

Supplementary figure 3

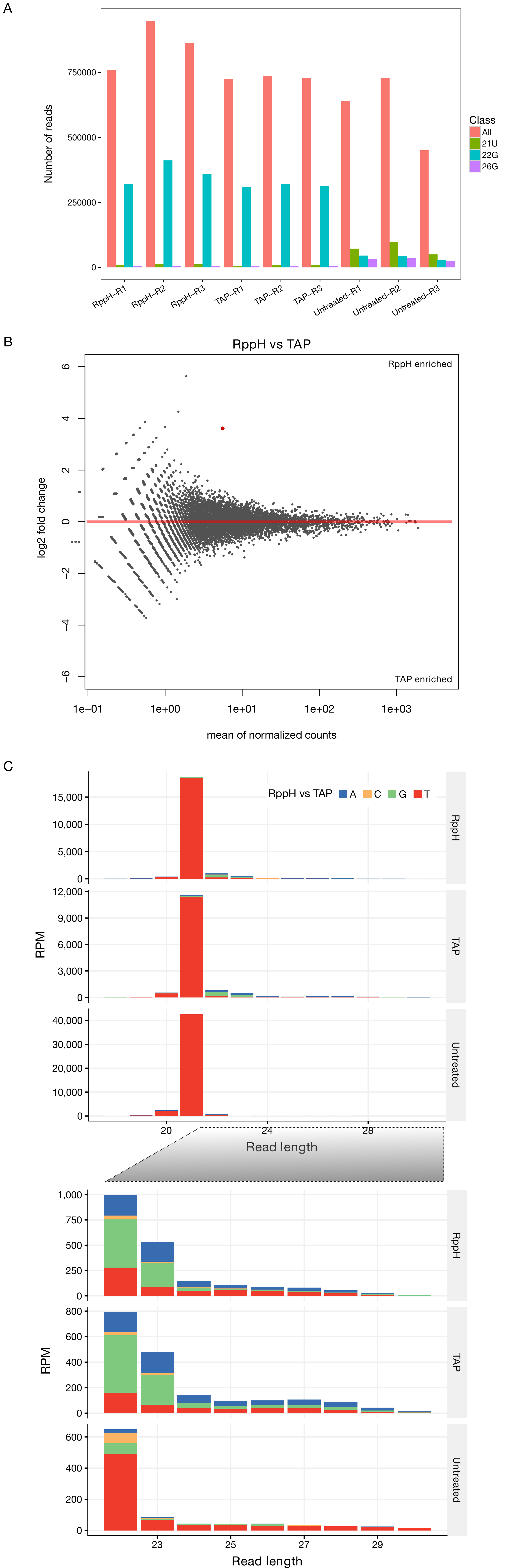

Supplement: Supplementary file 1 [file mmc1.pdf]
