# Supplementary material for: RppH can faithfully replace TAP to allow cloning of 5′-triphosphate carrying small RNAs
Source: MethodsX. 2019 Jan 29;6:265–72. doi: 10.1016/j.mex.2019.01.009 (PMC6369235; doi:10.1016/j.mex.2019.01.009)
Supplement: Supplementary file 2 [file mmc2.docx]

**Figure S1.** Per base sequence quality of TAP- and RppH-treated libraries.

**Figure S2.** Genomic coverage of 22G-RNAs in TAP- and RppH-treated libraries. All chromosomes are shown.

**Figure S3. 22G-RNA enrichment in 22G-RNA targets and 21U-RNA percursors capture in RppH-treament** . (A) Reads from both TAP- and RppH-treated libraries mapping to known 22G-RNA targets (see methods) are enriched for 22G-RNAs. (B) MA plot showing the DESeq2 results of 22G-RNA targeting. (C) Summary of the length and 5’ nucleotide bias of reads mapping to annotated 21U-RNAs, in RPM. The three bottom panels comprise an enlarged view of 22-30 nucleotide reads to better appreciate the features of putative 21U-RNA precursors.
